# Neonatal Hydrops and Biliary Atresia as an Early Presentation of Mevalonate Kinase Deficiency: A Case Report

**DOI:** 10.7759/cureus.107591

**Published:** 2026-04-23

**Authors:** Suzan A Almomani, Hafis Ibrahim C Ponnambath, Amal Yahya

**Affiliations:** 1 Neonatology, Corniche Hospital, Abu Dhabi, ARE

**Keywords:** biliary atresia, inborn errors of metabolism, mevalonate kinase deficiency, neonatal autoinflammatory disease, neonatal cholestasis, nonimmune hydrops fetalis, whole exome sequencing

## Abstract

Mevalonate kinase deficiency (MKD) is a rare autosomal recessive autoinflammatory disorder with a broad clinical spectrum. Neonatal presentation is uncommon and may mimic severe infection, leading to diagnostic delay. We report a preterm female infant who presented with non-immune hydrops fetalis and persistent systemic inflammation from the first week of life, despite repeated negative blood and CSF cultures and multiple courses of broad-spectrum antibiotics. The infant subsequently developed progressive cholestasis with pale stools, and biliary atresia was confirmed by hepatobiliary imaging and intraoperative cholangiography, followed by portoenterostomy. Extensive metabolic, immunologic, and infectious evaluations were unrevealing. Whole-exome sequencing identified compound heterozygous pathogenic variants in the *MVK* gene, confirming the diagnosis of MKD. Perinatal-onset MKD is exceptionally rare and often presents with non-specific systemic inflammation that can mimic neonatal sepsis, resulting in delayed diagnosis and significant diagnostic and therapeutic challenges. The infant was treated with supportive care, corticosteroids, and the IL-1 receptor antagonist anakinra, but the clinical course was complicated by progressive multiorgan failure, and she died at 3.5 months of age. This case highlights the diagnostic challenges of neonatal-onset MKD and suggests a possible association between MKD, hydrops fetalis, and biliary atresia that requires further investigation. Early consideration of autoinflammatory disorders and timely genetic testing may facilitate diagnosis and guide management in neonates with unexplained systemic inflammation.

## Introduction

Infections in newborn infants can be associated with mortality and severe morbidity. The clinical signs of infection can be non-specific in newborns, and clinicians often rely on inflammatory markers to make a presumptive diagnosis of sepsis. The routinely done markers in this age group include C-reactive protein (CRP), procalcitonin, and white cell counts [1]. These markers have variable sensitivity and specificity for diagnosing neonatal infection and should be interpreted in the clinical context [2]. However, they are very often used to make a diagnosis of culture-negative sepsis and to treat infants empirically with antibiotics, even in the absence of positive bacterial cultures [3]. Though this approach may be justified in most instances, extremely rarely, there can be other causes of raised inflammatory markers in infants, such as autoinflammatory syndromes [1].

Mevalonate kinase deficiency (MKD) is one such rare autosomal recessive autoinflammatory disorder caused by mutations in the *MVK* gene. Discovered in the 1980s, it encompasses a clinical spectrum ranging from the milder hyperimmunoglobulinemia D syndrome to the severe mevalonic aciduria [4-6]. Early diagnosis is often challenging due to non-specific symptoms and rarity. Perinatal-onset MKD is exceptionally rare and poses diagnostic and therapeutic dilemmas [5,6]. MKD results from impaired isoprenoid biosynthesis, which disrupts intracellular regulatory pathways and drives excess inflammasome activation, with increased production of proinflammatory cytokines, particularly IL-1. In severe early-onset disease, this uncontrolled systemic inflammation may contribute to capillary leak, hypoalbuminemia, hepatic dysfunction, and progressive multiorgan involvement. These mechanisms may plausibly contribute to fetal hydrops and postnatal cholestatic liver disease, although the exact pathophysiologic relationship remains incompletely understood. We report a case of neonatal-onset MKD, which presented diagnostic and therapeutic challenges.

## Case presentation

The proband was a preterm female infant born at 30+1 weeks’ gestation, weighing 1.67 kg, to a non-consanguineous couple of Indian origin. The mother was a 29-year-old previously healthy woman with a history of intrauterine fetal demise at 21 weeks in a prior pregnancy. During the index pregnancy, the 21-week anomaly scan demonstrated fetal growth restriction and mesenteric calcifications. Serial antenatal imaging later showed progression to non-immune hydrops fetalis with ascites and pleural effusions by 30 weeks’ gestation. Maternal evaluation for common infectious causes was negative, including TORCH screening. Non-invasive prenatal testing was low risk for common aneuploidies. Further antenatal genetic testing was offered but declined. Delivery occurred by emergency cesarean section because of abnormal cardiotocography. The baby required intubation and ventilation from birth.

**Figure 1:**
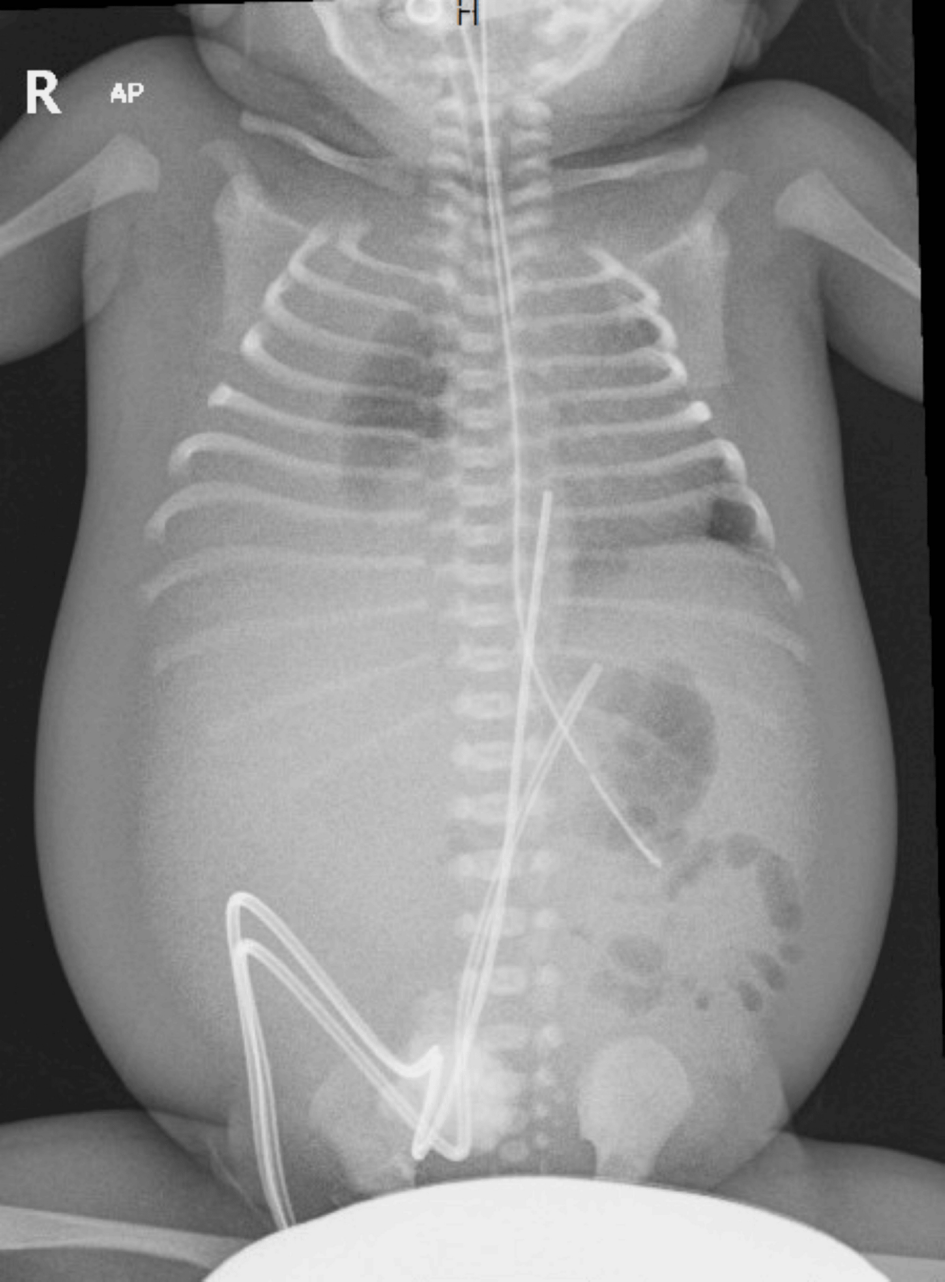
Postnatal chest and abdomen radiograph showing features of hydrops fetalis, including marked ascites.

**Figure 2:**
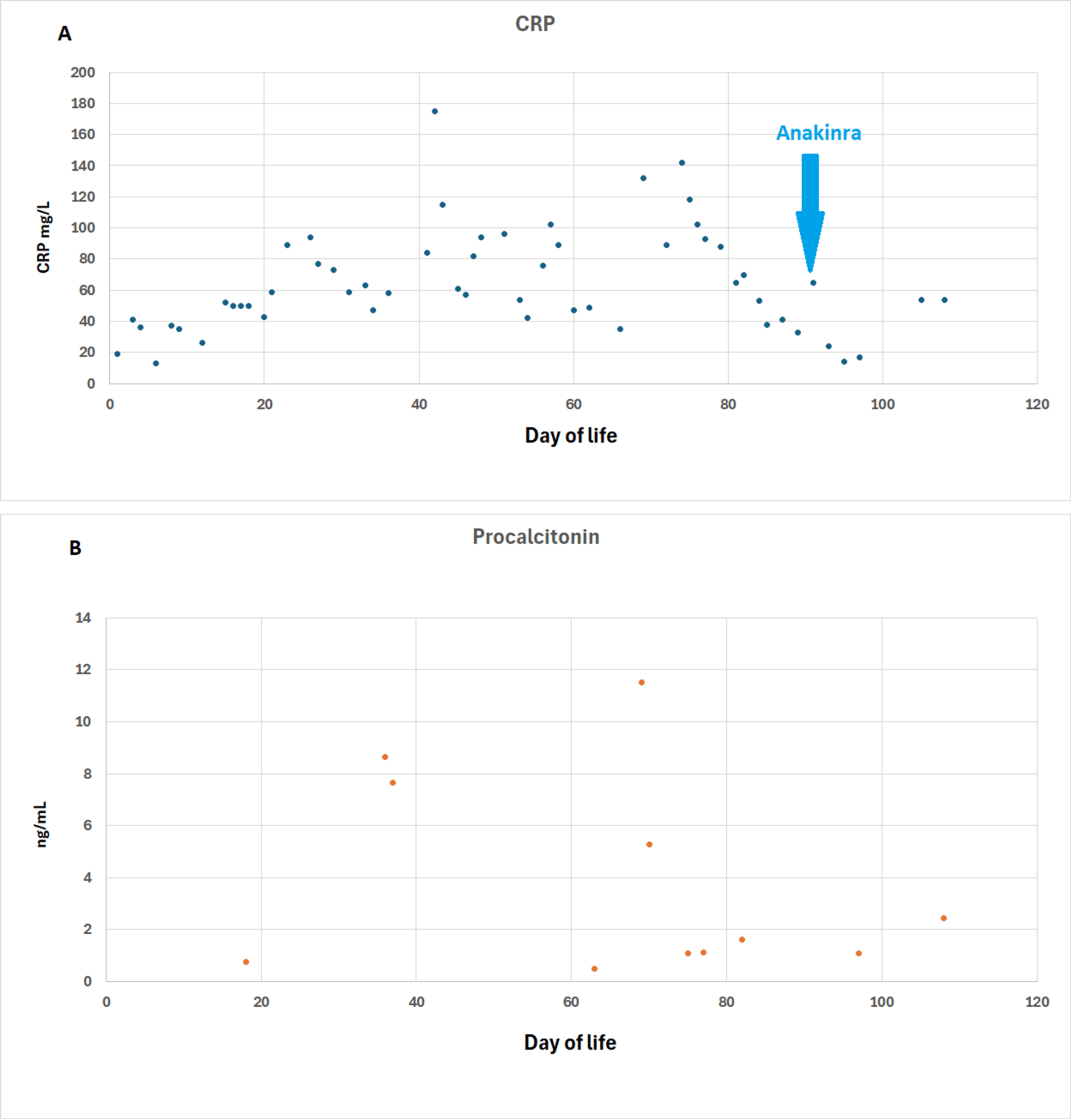
Trend of selected anti-inflammatory markers in infants (A) CRP and (B) procalcitonin

Postnatal physical examination revealed generalized edema and ascites as seen in Figure 1. Given the presence of non-immune hydrops fetalis, a broad etiologic evaluation was undertaken. Initial blood gas showed a pH of 7.23, bicarbonate of 22 mmol/L, base deficit of -2, glucose of 3.1 mmol/L, and lactate of 3 mmol/L. There was no significant metabolic acidosis, hypoglycemia, or hyperlactatemia at birth to specifically suggest an inborn error of metabolism at initial presentation. Total bilirubin was 89 μmol/L (direct bilirubin: 40 μmol/L) at 24 hours of life. The mother’s blood group was A positive, and the neonate’s blood group was A positive. Hematologic evaluation excluded immune hydrops, and there was no evidence of fetomaternal blood group incompatibility, significant fetal anemia, or fetomaternal hemorrhage. Cardiac evaluation, including echocardiography, did not demonstrate a structural cardiac lesion sufficient to explain the hydrops. Metabolic evaluation was also pursued and did not identify an alternative inherited metabolic disorder (Table 1). Collectively, these findings supported a diagnosis of non-immune hydrops of unclear etiology at birth. Initial laboratory tests showed elevated CRP with a normal white blood cell count and no other signs of sepsis. CRP remained elevated throughout the hospital stay despite antibiotic therapy (Figure 2).

**Table 1:** List of investigations performed for non-immune hydrops ANA, antinuclear antibody; Anti-dsDNA, anti-double-stranded DNA antibody; CMV, cytomegalovirus; ENA, extractable nuclear antigen; IgA, immunoglobulin A; IgG, immunoglobulin G; IgM, immunoglobulin M; Jo-1, histidyl-tRNA synthetase antibody (anti-Jo-1 antibody); MKD, mevalonate kinase deficiency; PCR, polymerase chain reaction; Sm, Smith antigen; SSA, Sjögren syndrome antigen A; SSB, Sjögren syndrome antigen B; TORCH, toxoplasmosis, other (including syphilis), rubella, cytomegalovirus, and herpes simplex virus.

Category	Investigation	Result	Reference range	Clinical interpretation
Metabolic	Triglycerides	1.21 mmol/L	0.31-1.41	Normal
	Ammonia	91 μmol/L	______	Normal for age
	Plasma amino acids	Normal profile	______	No evidence of aminoacidopathy
	Acylcarnitine	Normal	______	No fatty acid oxidation defect
	Urine organic acids	Mild elevation in lactic acid, detectable mevalonolactone, and mevalonic acid in minimal amounts	______	Suspicious for MKD, limited by dilution, repeat recommended
	Urine-reducing substances	Negative	______	No galactosemia
	Very long-chain fatty acids	Normal	______	No peroxisomal disorder
Immunologic	IgA	0.38 g/L	<0.83	Normal
	IgG	9.8 g/L	2.3-14.1	Normal
	IgM	0.74 g/L	<1.45	Normal
	ANA	Positive	______	Suggests immune activation
	Anti-dsDNA	<9.8 IU/mL	<26	Negative
	ENA panel (Sm, SSA, SSB, Jo-1, histone)	Negative	______	No specific autoimmune disease
	Complement C3	0.93 g/L	0.9-1.8	Normal
	Complement C4	0.08 g/L	0.1-0.4	Low
	Serum amyloid A	<6.4 mg/L	<6.4	Normal
	Ferritin	931 ng/mL	21-597	Elevated, suggestive of inflammation
Infectious	TORCH screen	Negative	______	No congenital infections
	Hepatitis B and C	Negative	______	Excluded
	Parvovirus	Negative	______	Excluded
	Urine CMV PCR	Not detected	______	No viral detection
	Blood group	A positive	______	______
	Direct antiglobulin test	Negative	______	No hemolysis
Screening	Newborn screen (tandem mass spectroscopy)	Negative	______	No common metabolic disorders detected

Given persistent elevation of inflammatory markers and recurrent clinical concern for neonatal sepsis despite negative cultures, the patient received empiric broad-spectrum antibiotics during multiple episodes of suspected infection, including penicillin, gentamicin, teicoplanin, amikacin, linezolid, meropenem, and vancomycin at various stages. These were initiated and progressively escalated to broader spectrum antibiotics due to ongoing systemic inflammation, prematurity, and the difficulty of excluding culture-negative neonatal sepsis. However, all blood, urine, and CSF cultures remained persistently negative throughout the course. The procalcitonin levels were intermittently high, and the serum amyloid A protein was within normal range. Investigations for immunodeficiencies, autoimmune disorders, and congenital infections were all negative (Table 1). All the metabolic investigations, including newborn screen by tandem mass spectroscopy, were normal apart from urine organic acid profile.

Urine organic acid analysis showed mildly elevated lactic acid with detectable mevalonolactone and minimal mevalonic acid; however, interpretation was limited by marked sample dilution. Although not diagnostic, these findings raised suspicion for MKD, and repeat sampling was recommended. 

**Figure 3:**
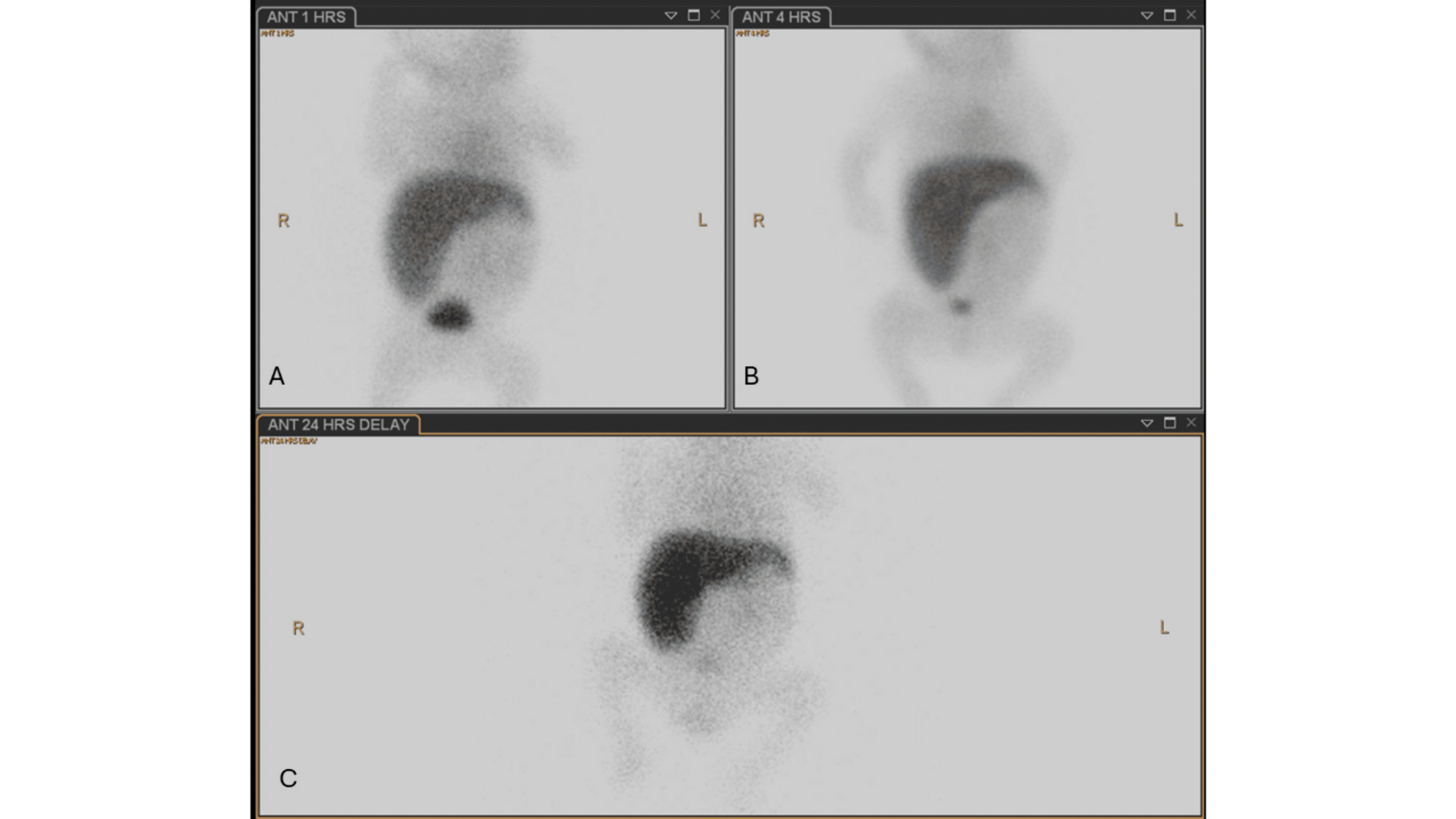
Hepatobiliary iminodiacetic acid (HIDA) scan demonstrating persistent hepatic uptake of radiotracer. (A) 1-hour image showing hepatic uptake without visualization of the biliary tree or bowel. (B) 4-hour delayed image demonstrating persistent hepatic tracer retention without excretion. (C) 24-hour delayed image showing continued hepatic uptake without bowel visualization, suggestive of biliary obstruction.

She was also noted to have cholestatic hyperbilirubinemia from birth, which progressively worsened with pale stools. Biliary atresia was confirmed by a radionuclide scan (Figure 3), followed by diagnostic laparoscopy and intraoperative cholangiogram. A Kasai portoenterostomy was subsequently performed at three months of age.

Further evaluation included whole-exome sequencing, which revealed autosomal recessive *MVK*-related disease due to the presence of compound heterozygous variants c.1097A>G (p.Asp366Gly) and c.577G>C (p.Glu193Gln) in the *MVK* gene, confirming the diagnosis of MKD. Parental testing was performed, the results of which came after the baby had unfortunately demised. The mother was a heterozygous carrier for c.577G>C (p.Glu193Gln) and the father for c.1097A>G (p.Asp366Gly). Thus, the variants are confirmed to be in trans, and the child was compound heterozygous. The baby also had an additional finding of heterozygous *MED12* gene mutation c.6521T>C (p.Phe2174Ser), suggestive of Hardikar syndrome, which may explain the biliary tree abnormalities described in this condition.

The patient was started on anti-inflammatory treatment, including corticosteroids and IL-1 receptor antagonist (anakinra), at a dose of 1 mg/kg/day administered subcutaneously once daily with poor response. Unfortunately, the baby continued to deteriorate with progressive multiorgan failure due to uncontrolled inflammation and passed away at the age of 3.5 months. 

## Discussion

Mevalonate kinase is an enzyme produced by the *MVK* gene, responsible for the production of cholesterol and other isoprenoids involved in various cellular functions. Mutations in this gene are inherited in an autosomal recessive pattern and lead to a deficiency of mevalonate kinase, causing a range of autoinflammatory disorders collectively known as MKD [4,5]. Patients often present with recurrent febrile episodes, lymphadenopathy, hepatomegaly, rash, arthralgia, and elevated inflammatory markers. MKD typically manifests during infancy or early childhood. However, perinatal and neonatal presentations, though exceedingly rare, can occur and are considered markers of severe disease within the spectrum [6-8]. Hydrops fetalis is a rare and atypical presentation of MKD [4].

This case highlights the importance of considering rare inflammatory or genetic conditions in the presence of persistent systemic inflammation after birth. MKD results in impaired isoprenoid synthesis, leading to dysregulation of the inflammatory cascade and excessive IL-1-mediated inflammation. This systemic inflammatory state may contribute to increased vascular permeability and capillary leak, potentially explaining the development of hydrops fetalis. Additionally, chronic inflammation and hepatocellular dysfunction may contribute to cholestasis. However, the association with biliary atresia remains uncertain and may represent a coincidental finding or a modifying genetic factor. In our case, the combination of elevated CRP, ferritin, and thrombocytopenia pointed toward an autoinflammatory process, eventually leading to the diagnosis of MKD through genetic testing. Whole-exome sequencing ultimately revealed pathogenic mutations in the *MVK* gene, confirming the diagnosis.

While a direct causal relationship between MKD and biliary atresia cannot be established from a single case, the presence of systemic inflammation may plausibly contribute to hepatobiliary dysfunction, warranting further investigation. Cholestasis is an uncommon but reported feature in MKD [9]. However, the co-presence of biliary atresia in our patient is unique and has not been well described in association with MKD. This patient also had a mutation in the *MED12* gene, which can be causative of Hardikar syndrome and could explain the cholestasis [10,11]. Hardikar syndrome is an X-linked dominant multiple congenital anomaly disorder reported only in females. Features include foregut malformations, intestinal malrotation, liver and biliary tract disease, genitourinary abnormalities, cleft lip and palate, and pigmentary retinopathy. However, the infant did not demonstrate other common features of the syndrome, such as cleft lip/palate or genitourinary abnormalities. Therefore, it remains unclear whether the biliary atresia in this infant is attributable to the *MED12* mutation or represents a separate or coincidental finding in MKD.

Management of MKD remains challenging, particularly in neonates with severe or atypical presentations. Prognosis is variable and depends on disease severity and treatment response. Treatment options in older children include anti-inflammatory agents such as corticosteroids, IL-1 receptor antagonists (e.g., anakinra), or biologics targeting the inflammatory cascade [4]. However, data regarding their use in neonates are extremely limited [7]. Early diagnosis, close monitoring, and timely immunomodulatory therapy -- when indicated -- may help mitigate long-term complications and improve outcomes.

Multidisciplinary care is essential, particularly in such rare and complex cases. In this case, supportive care and surgical intervention for biliary atresia were prioritized. Immunomodulatory therapy was initiated once the diagnosis became clear, though with limited response, reflecting the severity of presentation at such an early age. The family was referred for parental testing and genetic counseling.

## Conclusions

This case highlights a rare and severe neonatal presentation of MKD characterized by non-immune hydrops fetalis, persistent systemic inflammation, and biliary atresia. While a causal relationship cannot be established, this case suggests a possible association that warrants further investigation. It underscores the importance of considering autoinflammatory and metabolic disorders in a neonate with unexplained hydrops and ongoing inflammation, particularly when an additional *MED12* variant raises the possibility of a modifying or overlapping syndromic contribution to the observed phenotype. Persistent elevation of inflammatory markers in the absence of infection should prompt consideration of autoinflammatory and genetic disorders in the neonatal period. Early recognition and timely genetic testing are essential to guide management, inform prognosis, and support family counseling in such complex presentations.
